# The chemokine CXCL13 in cerebrospinal fluid in children with Lyme neuroborreliosis

**DOI:** 10.1007/s10096-018-3334-3

**Published:** 2018-08-06

**Authors:** Anna J. Henningsson, Malin Lager, Rebecka Brännström, Ivar Tjernberg, Barbro H. Skogman

**Affiliations:** 1 Clinical Microbiology, Division of Laboratory Medicine, Region Jönköping County, Sweden; 2 Department of Clinical and Experimental Medicine, Linköping University, Linköping, Sweden; 3 School of Medical Sciences, Örebro University, Örebro, Sweden; 4 Department of Clinical Chemistry and Transfusion Medicine Kalmar County Council, Linköping University, Linköping, Sweden; 5 Department of Pediatrics, Falun General Hospital, Falun, Sweden; 6 Center for Clinical Research (CKF) Dalarna, Uppsala University, Falun, Sweden

**Keywords:** CXCL13, Chemokine, Lyme neuroborreliosis, Diagnostic test, Cerebrospinal fluid, Children

## Abstract

Anti-*Borrelia* antibodies in the cerebrospinal fluid (CSF) are required for definite diagnosis of Lyme neuroborreliosis (LNB). However, children often present with early LNB, and antibody production in the CSF may not be demonstrated. Recent studies have suggested the chemokine CXCL13 to be an early marker for LNB. The aim of the study was to evaluate CXCL13 for laboratory diagnosis in pediatric LNB patients and to evaluate the association with pleocytosis in CSF, clinical features, and recovery. CSF samples were collected from LNB patients, classified as definite LNB (*n* = 44) or possible LNB (*n* = 22), and controls classified as non-LNB (*n* = 102) or other specific diagnoses (*n* = 23). CSF samples were analyzed with the recomBead CXCL13 assay (Mikrogen Diagnostik, Germany), cut-off 160 pg/mL. CXCL13 was significantly higher in LNB patients compared to controls (*p* < 0.001). Among LNB patients, 58/66 had elevated CXCL13, and among controls, 111/125 had CXCL13 levels under cut-off (sensitivity 88%, specificity 89%). In LNB patients with pleocytosis but no detectable anti-*Borrelia* antibodies in CSF (possible LNB), CXCL13 was elevated in 16/22 (73%). A weak correlation between CXCL13 and pleocytosis in CSF was found in LNB patients (Rho = 0.46, *p* < 0.01), but no differences in CXCL13 levels in relation to specific clinical features. In conclusion, CXCL13 is elevated in CSF in children with LNB, showing acceptable sensitivity and specificity. In patients with possible LNB, CXCL13 was elevated in a majority of cases (73%) and is suggested as a complementary diagnostic tool in pediatric LNB patients. CXCL13 was not associated with specific clinical features or recovery.

## Introduction

Lyme borreliosis (LB) is a tick-borne infection caused by spirochetes belonging to the *Borrelia burgdorferi* sensu lato complex [1]. The main human pathogens are *Borrelia* (*B.*) *garinii*, *B. afzelii*, and *B. burgdorferi* sensu stricto [2]. The *Borrelia* spirochetes are invasive and motile, easily disseminating through different tissues in the body. These bacteria do not produce any toxins but they activate the host’s immune defense, causing numerous inflammatory responses that in turn generate the symptoms of the disease [3, 4].

Lyme neuroborreliosis (LNB) is caused by dissemination of the spirochetes (most frequently *B. garinii*) into the central nervous system (CNS), leading to inflammation in the CNS (meningitis) and affection of cranial or peripheral nerves [3, 5, 6].

The clinical features of LNB differ somewhat between children and adults [5, 7, 8]. In children, the most common manifestations are acute facial nerve palsy and/or subacute meningitis [5, 7]. Less specific but common symptoms are headache, fatigue, nausea, loss of appetite, or unspecific pain [9]. Since none of these symptoms are pathognomonic for LNB, laboratory tests are needed to confirm the diagnosis [3].

European guidelines state three criteria for the diagnosis of definite LNB: (i) neurological symptoms attributable to LNB, (ii) pleocytosis in cerebrospinal fluid (CSF), and (iii) intrathecally produced anti-*Borrelia* antibodies [10]. However, the intrathecal antibody production does not start immediately after dissemination to the nervous system and as a consequence, anti-*Borrelia* antibodies may still be absent if lumbar puncture is performed early in the course of the disease [11]. Children often present with early LNB and short duration of neurological symptoms, making the test based on intrathecal anti-*Borrelia* antibody production less sensitive in this group of patients [12, 13].

The chemokine CXC motif ligand 13 (CXCL13) is known primarily for its function as a B cell- and follicular T-helper cell-attractant in lymphoid tissues [14], but CXCL13 is also produced in non-lymphoid tissues during inflammation, where it functions primarily as an attractor of B cells [14–16]. B cells are the source of antibody production and must therefore migrate into the CNS before intrathecal antibody production can be initiated [15]. Consequently, the chemokine CXCL13 has been suggested as a candidate for a possible marker for early LNB [17], and previous studies have indicated that intrathecal CXCL13 levels are generally significantly more elevated in LNB as compared to many other CNS conditions [18, 19].

CXCL13 also seems to be a valid clinical marker for active *Borrelia*-induced inflammation, and it has been shown in several studies that the CXCL13 concentration is high during active inflammation and decreases rapidly after successful antibiotic treatment of LNB [18, 20, 21]. It has also been suggested that CXCL13 in the CSF may be a marker for disease duration [21]. Different methods and cut-off levels have been suggested in different studies [18, 19, 22–25], but no consensus has been established so far.

Since children often present with early LNB, and anti-*Borrelia* antibodies may not yet be present in the CSF, it is of importance to evaluate the usefulness of complementary methods for diagnostic purposes in this group of patients, and CXCL13 has been a promising candidate [19, 22]. Furthermore, it is of interest to investigate whether the concentration of CXCL13 in CSF correlates with certain clinical features, such as duration of symptoms or clinical recovery in order to better understand the role of CXCL13 in relation to symptomatology, pathogenesis, disease course, and prognosis of LNB [4, 19].

The aim of the study was to evaluate the usefulness of CXCL13 for laboratory diagnosis in well-characterized pediatric LNB patients and controls, and to evaluate the association to pleocytosis in CSF, clinical features, and recovery.

## Materials and methods

### Patients and controls

Children with suspected LNB were recruited from seven pediatric clinics in a Lyme endemic area in southeast Sweden during the period 2010 to 2014. Patients were clinically evaluated on admission and underwent a lumbar puncture as part of the routine investigation. Additional blood and CSF samples were taken at the same occasion for research purposes. A questionnaire was completed by children and parents/guardians, including questions regarding clinical symptoms, observed tick bites or erythema migrans, previous antibiotic treatment, and the overall health of the child. All children in the study were followed clinically for 2 months, and a questionnaire for self-reported symptoms was employed.

### Classification of LNB patients and controls

Classification of LNB patients was made according to European guidelines [10]. Definite LNB was defined as (i) symptoms attributable to LNB, (ii) pleocytosis in CSF, and (iii) intrathecally produced anti-*Borrelia* antibodies. Possible LNB was defined as (i) symptoms attributable to LNB, (ii) pleocytosis in CSF, or (iii) intrathecally produced anti-*Borrelia* antibodies. All children in the possible LNB group in this study had pleocytosis in CSF but no detectable intrathecally produced anti-*Borrelia* antibodies, and no clinical signs or laboratory evidence for other infection.

All patients classified as definite LNB and possible LNB received antibiotics according to national guidelines (i.e., ceftriaxone i.v. 50–100 mg/kg once daily for 10–14 days for children < 8 years of age, and doxycycline p.o. 4 mg/kg once daily for 10–14 days for children ≥ 8 years of age). All patients responded well to treatment.

Definite LNB (*n* = 44) and possible LNB (*n* = 22) patients are, in this study, referred to as LNB patients (*n* = 66).

At the 2-month follow-up, children were evaluated as recovered/not recovered based on history, response to treatment, clinical findings, and self-reported persistent symptoms.

Two control groups were included in the present study. The first control group consisted of patients classified as non-LNB cases (*n* = 102). These patients presented with neurological symptoms attributable to LNB but with no pleocytosis in CSF and no intrathecally produced anti-*Borrelia* antibodies. Non-LNB patients were mainly patients with idiopathic facial nerve palsy or headache of unknown origin. No patient in the non-LNB group received any other specific neurological diagnosis.

The second control group consisted of children with other specific diagnoses (*n* = 23), such as viral meningitis (*n* = 4), epilepsy (*n* = 4), idiopathic intracranial hypertension (*n* = 3), post infectious encephalitis (*n* = 2), infantile spasm (*n* = 2), migraine (*n* = 2), head trauma (*n* = 1), stroke (*n* = 1), attention deficit hyperactivity disorder (ADHD) (*n* = 1), neurofibromatosis type 1 (*n* = 1), and Guillain-Barre syndrome (*n* = 1).

Some patients in the group of other specific diagnoses presented with pleocytosis in CSF (viral meningitis *n* = 4 and post infectious encephalitis *n* = 2). They all had specific clinical symptoms associated to either viral meningitis (high fever, headache, neck stiffness, photophobia) or post infectious encephalitis (viral infection followed by confusion and seizures), but only one patient with viral meningitis had a specific causative agent (enterovirus) confirmed by PCR. All patients with other specific diagnoses were negative for intrathecally produced anti-*Borrelia* antibodies, including the six patients with pleocytosis. Patients classified as non-LNB cases (*n* = 102) and other specific diagnoses (*n* = 23) are being referred to in this study as controls (*n* = 125).

Clinical characteristics of patients in different groups are shown in Table 1.

**Table 1:** Characteristics of LNB patients and controls

On admission		Definite LNB (*n* = 44)	Possible LNB (*n* = 22)	Non-LNB (*n* = 102)	Other diagnoses (*n* = 23)
Age	Median (range)	6 (2–11)	8 (4–13)	13 (1–19)	10 (1–16)
Sex	Female, *n* (%)	19 (43)	10 (45)	66 (65)	12 (52)
Observed tick bite	Yes, *n* (%)	27 (61)	11 (50)	45 (44)	2 (9)
Duration of symptoms	< 1 week, *n* (%)	18 (41)	12 (55)	31 (31)	7 (30)
	1–4 weeks, *n* (%)	22 (50)	7 (32)	20 (20)	3 (13)
	1–2 months, *n* (%)	0 (0)	0 (0)	6 (6)	2 (9)
	> 2 months, *n* (%)	1 (2)	1 (5)	24 (24)	3 (13)
Clinical features	Facial palsy, *n* (%)	28 (64)	18 (82)	35 (34)	1 (4)
	Meningeal symptoms, *n* (%)	33 (75)	19 (86)	79 (77)	17 (74)
	Fatigue, *n* (%)	39 (89)	13 (59)	69 (68)	10 (43)
	Nausea and/or loss of appetite, *n* (%)	26 (59)	10 (45)	35 (34)	5 (22)
	Radiating pain (limbs)	18 (41)	7 (32)	14 (14)	1 (4)
	EM and/or lymphocytoma, *n* (%)	17 (39)	5 (23)	16 (16)	0 (0)
Laboratory findings	Pleocytosis ^**#**^, median (range)	162 (20–890)	50 (8–486)	0 (0–4)	0 (0–634)
	Anti-*Borrelia* antibodies in CSF ^**&**^ (IgG and/or IgM), *n* (%)	44 (100)	0 (0)	0 (0)	0 (0)
	Anti-*Borrelia* antibodies in serum (IgG and/or IgM), *n* (%)	24 (55)	14 (63)	22 (22)	1 (4)
Recovery at follow-up	Yes, *n* (%)	38 (86)	18 (82)	80 (78)	13 (57)

*LNB*, Lyme neuroborreliosis; *CSF*, cerebrospinal fluid; *EM*, erythema migrans; *IgG*, immunoglobulin G; *IgM*, immunoglobulin M; meningeal symptoms: headache, neck stiffness, and neck pain; ^#^pleocytosis > 5 × 10^6^ cells/L in CSF; ^&^intrathecally produced anti-*Borrelia* antibodies (IDEIA Lyme neuroborreliosis kit) (26)

### Laboratory analysis

All patients were analyzed for intrathecal anti-*Borrelia* antibody production (IgG and/or IgM) as part of the clinical routine evaluation on admission at each pediatric department, all using the flagella antigen-based enzyme-linked immunosorbent assay (ELISA), IDEIA Lyme Neuroborreliosis Kit (Oxid, Hampshire, UK) [26]. An index > 0.3 was considered as positive, indicating intrathecal production of anti-*Borrelia* antibodies according to the manufacturer’s instructions. Data from anti-*Borrelia* antibodies in serum was not separately available for patients with positive index with the IDEIA assay, noted as NA (not available) in Table 1. Pleocytosis in CSF was analyzed as part of routine clinical evaluation and defined as > 5 × 10^6^ cells/L [27].

CSF and blood samples for CXCL13 analyses were taken before the start of antibiotic treatment and stored at − 70° until analyses were performed at the Laboratory of Clinical Microbiology, Division of Laboratory Medicine, and Region Jönköping County, Sweden. The CXCL13 analyses in CSF were performed with the recomBead CXCL13 assay (Mikrogen Diagnostik GmbH, Neuried, Germany), a multiplex bead array, using Luminex xMAP technology. The software Xponent version 33.1.971.0 (Luminex Corporation, Austin, TX, USA) was used. The platform was applied according to the manufacturer’s instructions for evaluation of CXCL13 in CSF and 9 pg/mL was set as the lowest detection level. The cut-off level of 160 pg/mL was chosen according to previous studies [20, 21].

### Statistics

IBM SPSS Statistics, version 21 (IBM Corporation, USA) and Microsoft Excel 2016 (Microsoft Corporation, USA) were used for statistical analyses and illustrations.

Chi-squared test and Fisher’s exact test were used for non-continuous data. For non-parametric analysis, the Mann–Whitney *U* test was used when comparing continuous data between groups. Correlations were calculated using the Spearman’s rank-sum test using the correlation coefficient Rho when needed. A *p* value < 0.05 was considered statistically significant.

## Results

### Concentrations of CXCL13 in CSF in different patient groups

**Fig. 1:**
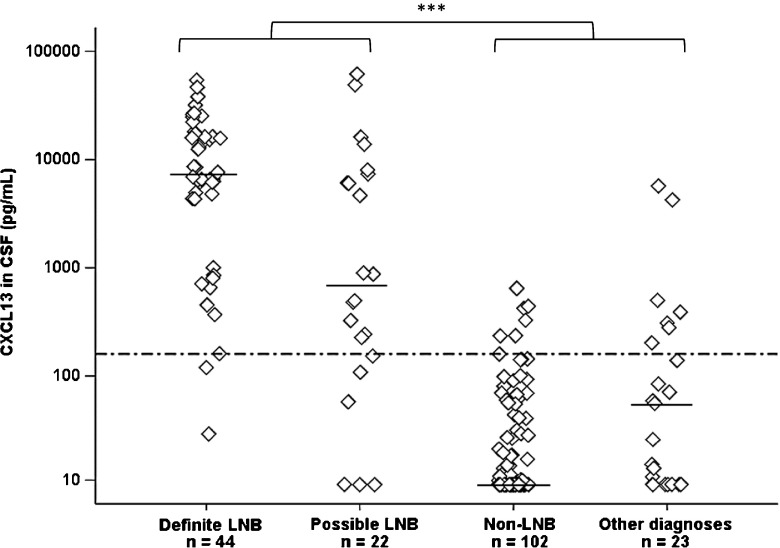
Concentration of CXCL13 (pg/mL) in patients with definite Lyme neuroborreliosis (LNB) (*n* = 44), possible LNB (*n* = 22), non-LNB (*n* = 102), and other specific diagnoses (*n* = 23). Horizontal bars represent median values and the dotted line shows the cut-off value at 160 pg/mL. Mann–Whitney *U* test was used to analyze the difference between LNB patients (definite and possible LNB) and controls (non-LNB and other diagnoses) (***p < 0.001)

The concentrations of CXCL13 in CSF ranged between 29 and 54,266 pg/mL (median 7303) in the definite LNB group, whereas levels of CXCL13 ranged between 9 and 61,643 pg/mL (median 688) in the possible LNB group (Fig. 1). Among non-LNB patients, CXCL13 ranged between 9 and 646 pg/mL (median 9), and in children with other specific diagnoses between 9 and 5746 pg/mL (median 56) (Fig. 1). The difference in CXCL13 concentrations between LNB patients (definite LNB and possible LNB) and controls (non-LNB and other specific diagnoses) was statistically significant (*p* < 0.001).

When comparing CXCL13 concentrations over and under the cut-off 160 pg/mL (i.e., calculation of positive versus negative test results) in LNB patients and controls, a statistically significant difference was found (*p* < 0.001). Among LNB patients, 58 out of 66 had a positive test result, giving the CXCL13 recomBead test in CSF a sensitivity of 88%. Calculations from controls showed a negative test result in 111 out of 125, giving the CXCL13 recomBead test in CSF a specificity of 89%.

### Concentrations of CXCL13 in CSF in patients with possible LNB compared to children with other specific diagnoses with pleocytosis in CSF

**Fig. 2:**
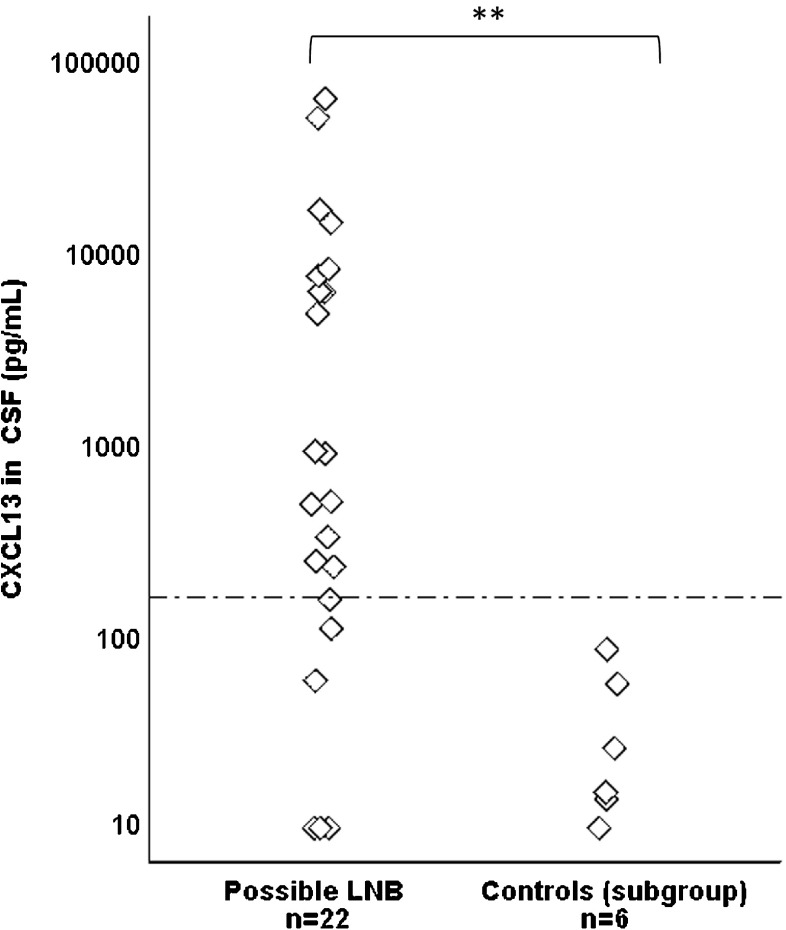
Concentrations of CXCL13 (pg/mL) in patients with possible Lyme neuroborreliosis (LNB), and patients with other specific diagnoses (subgroup of controls with pleocytosis). The dotted line shows the cut-off at 160 pg/mL. Mann–Whitney *U* test was used to analyze differences between groups (**p < 0.01)

To be able to evaluate whether CXCL13 may be helpful in excluding LNB in children with neurological manifestations and pleocytosis caused by other etiologies, the two groups of children with these properties were examined more closely. Among LNB patients, all 22 children with possible LNB presented with neurological symptoms and pleocytosis in CSF, but no detectable intrathecally produced *Borrelia* antibodies. Among children with other specific diagnoses, six patients presented with neurological symptoms and pleocytosis in CSF; viral meningitis (*n* = 4) and post-infectious encephalitis (*n* = 2). The CXCL13 concentrations in these two groups of children are shown in Fig. 2.

There was a statistically significant difference in CXCL13 concentrations between patients with possible LNB (*n* = 22), and patients in the subgroup of controls with other specific diagnoses and pleocytosis (*n* = 6) (*p* < 0.01) (Fig. 2). There was also a significant difference between the two groups when comparing the CXCL13 levels over and under the cut-off (*p* < 0.001). All patients in the subgroup of controls with other specific diagnoses and pleocytosis (*n* = 6) had CXCL13 levels under the cut-off (Fig. 2).

Based on the cut-off 160 pg/mL, 16 of the 22 patients (73%) with possible LNB were CXCL13 positive and six were CXCL13 negative. Laboratory findings of these six CXCL13 negative patients in the possible LNB group are shown in Table 2. Levels of CSF cell counts were generally low, all patients had short duration of symptoms and two patients were anti-*Borrelia* IgM-antibody positive in serum (Table 2). No patient had observed any EM but one had a *Borrelia* lymphocytoma on the left earlobe (no. 5).

**Table 2:** Clinical and laboratory findings in children with possible LNB and CXCL13 levels under cut-off (160 pg/mL) in CSF

Patient no	Duration of symptoms	CXCL13 (pg/mL)	Serum antibodies IgM/IgG	Total cells × 10^6^/L in CSF	EM/lymphocytoma
1	1–2 days	9	−/−	8	−/−
2	1–2 days	9	−/−	46	−/−
3	3–6 days	9	−/−	8	−/−
4	1–2 days	57	+/−	8	−/−
5	3–6 days	108	+/−	8	−/+
6	3–6 days	154	−/−	27	−/−

*LNB*, Lyme neuroborreliosis; *CSF*, cerebrospinal fluid; *EM*, erythema migrans; *IgM*, immunoglobulin M; *IgG*, immunoglobulin G

### Non-LNB patients with elevated CXCL13 in CSF

Out of all non-LNB patients (*n* = 102), seven children had CXCL13 concentrations over cut-off (Fig. 1). These patients are more closely described concerning clinical features and laboratory findings in Table 3. The CXCL13 concentrations were relatively low (161–646 pg/mL) and the durations of symptoms varied, ranging from 1 to 2 days, to over 2 months. One child reported a previous EM, four had observed tick bites and one patient had anti-*Borrelia* IgG-antibodies in serum (Table 3). One patient (no. 4) reported persistent mild facial nerve palsy at the 2-month follow-up, but the rest of the non-LNB patients with elevated CXCL13 in CSF were all recovered at follow-up.

**Table 3:** Clinical and laboratory findings in non-LNB patients with no pleocytosis but with elevated CXCL13 in CSF

Patient no	Diagnosis	Duration of symptoms	CXCL13 (pg/mL)	Serum antibodies IgM/IgG	Observed tick bite	EM
1	Idiopathic facial nerve palsy	1–2 weeks	161	−/+	Yes	No
2	Idiopathic facial nerve palsy	1–2 days	236	−/−	No	No
3	Headache (unspecified)	Unknown	237	−/−	Yes	No
4	Idiopathic facial nerve palsy	3–6 days	330	−/−	Yes	No
5	Impaired hearing	> 2 months	424	−/−	Yes	Yes
6	Idiopathic facial nerve palsy	1–2 weeks	446	−/−	No	No
7	Headache (unspecified)	> 2 months	646	−/−	No	No

*LNB*, Lyme neuroborreliosis; *CSF*, cerebrospinal fluid; *IgM*, immunoglobulin M; *IgG*, immunoglobulin G; elevated CXCL13 > 160 pg/mL

### Patients with other specific diagnoses and elevated CXCL13 in CSF

Seven patients with other specific diagnoses had elevated CXCL13 in CSF (204–5746 pg/mL) (Fig. 1). These patients had no pleocytosis in CSF, no anti-*Borrelia* antibodies in serum and no observed EM. Clinical features, diagnoses, and laboratory findings are shown in Table 4.

**Table 4:** Clinical and laboratory findings in children with other specific diagnoses and elevated CXCL13 in CSF

Patient no	CXCL13 (pg/mL)	Diagnosis	Pleocytosis ^**#**^	Serum antibodies IgM/IgG	EM
1	204	Idiopathic intracranial hypertension	No	−/−	No
2	281	Infantile spasm (untreated)	No	−/−	No
3	310	Idiopathic intracranial hypertension	No	−/−	No
4	445	Epilepsy (untreated)	No	−/−	No
5	502	Infantile spasm (untreated)	No	−/−	No
6	4270	Epilepsy (untreated)	No	−/−	No
7	5746	Neurofibromatosis type 1 (untreated)	No	−/−	No

*LNB*, Lyme neuroborreliosis; *CSF*, cerebrospinal fluid; *IgM*, immunoglobulin M; *IgG*, immunoglobulin G; ^**#**^pleocytosis > 5 × 10^6^ cells/L in CSF; elevated CXCL13 > 160 pg/mL

### Correlation between CXCL13 and pleocytosis in CSF

**Fig. 3:**
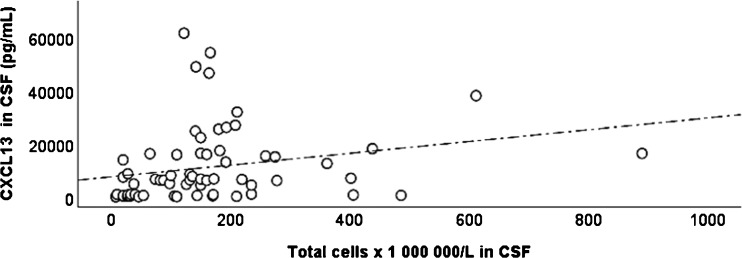
Concentrations of CXCL13 (pg/mL) plotted with total cell count in CSF. Spearman correlation test was used for statistical analysis. The correlation coefficient Rho = 0.46 (*p* < 0.01)

The CXCL13 concentrations of LNB patients were analyzed for correlation with the degree of pleocytosis in CSF (Fig. 3). A moderate positive correlation was found between CXCL13 levels and the total cell count in CSF, (Rho = 0.46, *p* < 0.01).

### CXCL13 and the associations to clinical features and recovery in LNB patients

**Fig. 4:**
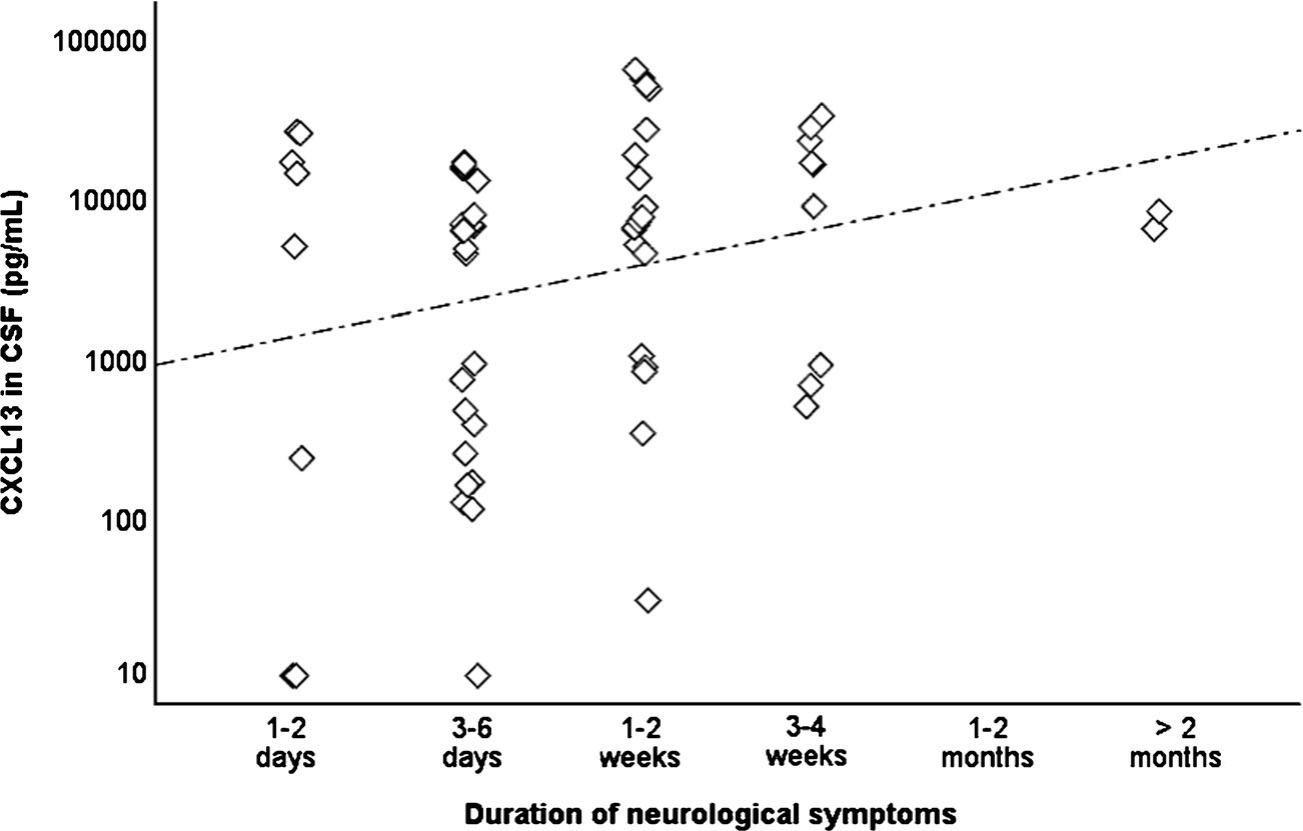
The concentration of CXCL13 (pg/mL) in the CSF of children with different duration of symptoms. Spearman correlation test was used for statistical analysis. The correlation coefficient Rho = 0.25 (*p* < 0.05)

The CXCL13 concentrations in CSF were analyzed for correlation with the duration of symptoms in LNB patients on admission (Fig. 4). A very weak positive correlation was found between higher CXCL13 levels and longer duration of symptoms (Rho = 0.25, *p* < 0.05). No differences in CXCL13 concentrations were found in relation to age, gender, observed tick bites, EM, or other clinical features. In addition, CXCL13 concentrations (on admission) were not more elevated in children who later reported persistent symptoms at the 2-month follow-up compared to children who were fully recovered at follow-up.

## Discussion

In this present study, the concentrations of CXCL13 in CSF were significantly higher in LNB patients compared to controls. These results are consistent with those observed in several previous studies [18–20, 22, 28, 29], all supporting the hypothesis that elevated concentrations of CXCL13 in CSF can be used as a marker for LNB. However, the overall sensitivity (88%) and overall specificity (89%) observed in our study were moderate, compared to previous studies on CXCL13 in CSF with sensitivities ranging between 88 and 100% and specificities between 89 and 100% [18, 19, 22, 25, 29, 30]. The variation in diagnostic performances between these studies may partly be explained by differences in classification of LNB patients and controls, the use of different laboratory methods with different intra-assay variability, and variations in cut-off levels for CXCL13 (ranging between 55 and 415 pg/mL) [18, 19, 25, 29]. The cut-off level used in the present study was chosen based on previous research on pediatric patients with definite LNB and non-LNB controls, analyzed with recomBead CXCL13 in CSF [30]. As shown in Fig. 1, the cut-off of 160 pg/mL seems to be appropriate also for this present pediatric material. The cut-off level of 160 pg/mL is also in consistency with the recommended cut-off level in a recently published meta-analysis [29].

When looking at the definite LNB group exclusively in our study, the sensitivity of CXCL13 in CSF was excellent at 95%, which is in line with previous studies [22, 28, 29]. More interestingly, the sensitivity in the possible LNB group was as high as 73%, similar to other studies but with one exception; a study with a comparable group of possible LNB patients in which the sensitivity of CSF-CXCL13 was found to be as low as 27% (despite the cut-off level 55 pg/mL) [19]. In that study, according to the authors, patients may have been pre-treated with antibiotics before CSF sampling, which is a possible weakness due to the retrospective study design [19]. Another study has previously found that CXCL13 levels elevated in CSF in LNB patients decrease rapidly after initiation of antibiotic treatment [21].

One can always discuss whether patients classified as possible LNB according to European guidelines [10] are true LNB patients without intrathecal antibody production, or if they are patients with other undiagnosed infections or conditions. Previously, evidence for undiagnosed other infectious diagnoses has not been found among these possible LNB patients [30]. In our present study, all children in the possible LNB group with elevated CXCL13 had pleocytosis with mononuclear dominance suggestive for LNB [27] and, in a majority of cases, facial nerve palsy and/or anti-*Borrelia* antibodies in serum and/or EM/ *Borrelia* lymphocytoma. No patient in the possible LNB group had clinical signs or laboratory evidence for other infections and all responded well to antibiotic treatment, which further supports the LNB diagnosis.

Among CXCL13 negative children in the possible LNB group (*n* = 6) (Table 2), pleocytosis was generally low and the LNB diagnosis could admittedly be questioned. However, two of these patients presented with acute facial nerve palsy and anti-*Borrelia* IgM antibodies in serum (one with a *Borrelia* lymphocytoma on the ipsilateral side) possibly indicating two very early LNB patients with pleocytosis but with no intrathecally produced anti-*Borrelia* antibodies and no CXCL13 in CSF at the time of lumbar puncture. All six patients responded well to antibiotic treatment, but at follow-up, one patient had mild persistent facial nerve palsy and one reported persistent headache. Taken all together, results from our study clearly support the hypothesis that CXCL13 analysis can be useful as diagnostic tool in early LNB when intrathecally produced anti-*Borrelia* antibodies are not yet detectable [19, 23, 29, 30].

In addition, we wanted to investigate patients in the non-LNB group with CXCL13 concentrations in CSF over cut-off in order to understand whether they could possibly be overlooked patients with very early LNB (Table 3). The majority of these patients had experienced symptoms for more than 1 week, only one reported an EM, and none had anti-*Borrelia* IgM in serum. Two patients had facial nerve palsy with a duration of 1–6 days on admission and CXCL13 concentrations in CSF above cut-off (236 and 330 pg/mL) but none of them had EM or anti-*Borrelia* IgM in serum to support a possible very early LNB diagnosis. No patient was later reinvestigated for prolonged symptoms. Thus, our current data does not support the hypothesis that non-LNB patients with elevated CXCL13 in CSF are patients with potential very early LNB, but this aspect should be further investigated.

In the group of children with other specific diagnoses, seven children had elevated CXCL13 in CSF (cut-off 160 pg/mL) (Table 4). Levels were moderately elevated, but two patients (one with epilepsy and one with neurofibromatosis type 1) had highly elevated CXCL13 (4270 and 5746 pg/mL respectively). None of the seven patients had pleocytosis in CSF, anti-*Borrelia* antibodies in serum or CSF, or had observed any tick bite or EM. These patients are most probably true or false positives. Interestingly, among children with other specific diagnoses and pleocytosis in CSF (viral meningitis *n* = 4 and post infectious encephalitis *n* = 2), no patient had elevated CXCL13 in CSF. These findings are in line with previous studies on CXC13 and well defined controls with other inflammatory or infectious diagnoses [18, 19, 24].

Our results suggest that CXCL13 could be used to support the LNB diagnosis in patients with clinical manifestations attributable to LNB and pleocytosis in CSF, but no intrathecally produced anti-*Borrelia* antibodies. However, interpretation of CXCL13 results should always be made in concordance with other laboratory tests and the clinical picture of the specific patient [19, 22, 30].

A moderate positive correlation between CXCL13 concentrations in CSF and the degree of pleocytosis in CSF (*p* < 0.01) was found in our study, but results are contradictory to previous studies where no significant correlation has been found [18, 28]. However, a correlation seems reasonable since CXCL13 is suggested to play a key role in migration of B cells into the CNS in LNB [15, 16]. In addition, the rise in cell count in CSF is followed by the onset of antibody production, which is seemingly logical considering that B cells are the source of intrathecally produced anti-*Borrelia* antibodies [16]. This correlation seems to further support the view that CXCL13 concentrations in CSF can be useful when evaluating children with suspected LNB, in cases when antibody production is not yet present. Furthermore, applying a linearized cut-off for evaluation of CXCL13 (i.e., using an algorithm in which both the level of the CXCL13 and the pleocytosis in the specific patient is included), as suggested by Markowicz et al. [31], could be an interesting way to further improve the diagnostic performance of CXCL13 in LNB.

Lastly, CXCL13 concentrations were analyzed for possible associations to specific clinical features. In a previous study performed on adult patients, a negative correlation was found between CXCL13 concentration in CSF, and duration of neurological symptoms reported on admission, with higher concentrations early in the course of the disease and lower concentrations later [21]. In our present study, the opposite was found, slightly indicating a higher concentrations of CXCL13 with longer duration of symptoms on admission (Rho 0.254, *p* < 0.05). However, in a previous study on pediatric patients, we found no significant correlation between duration of symptoms on admission and levels of CXCL13 in CSF [28]. Differences between studies on adult LNB patients compared to pediatric LNB patients could possibly be explained by general biological differences between children and adults, by different laboratory methods or cut-off levels for measuring CXCL13 concentrations in CSF, or by differences in duration of symptoms reported on admission (which is much longer in adults than in children). Again, one has to keep in mind that the role for CXCL13 in immune regulation and pathophysiology for LNB in both adults and pediatric patients is still largely unknown [4, 14]. Our results could possibly contribute small pieces of knowledge to help us come closer to understanding LNB pathogenesis in humans.

Finally, no differences in CXCL13 levels were found in relation to age, gender, observed tick bites, EM, or other clinical manifestations in the present study or in a previous study [28]. Furthermore, CXCL13 concentrations (on admission) were not elevated in children who later reported persistent symptoms at the 2-month follow-up, suggesting that CXCL13 in CSF could not be used as a predictive test for clinical recovery.

## Conclusion

The chemokine CXCL13 is elevated in CSF in children with LNB, showing a high sensitivity in the definite LNB group and an acceptable specificity. In patients with pleocytosis, but no intrathecally produced anti-*Borrelia* antibodies (possible LNB), the CXCL13 concentrations ware elevated in a majority of patients (73%) and CXCL13 is therefore suggested as a useful complementary diagnostic tool in pediatric patients. CXCL13 concentrations correlated moderately to the degree of pleocytosis in CSF and the duration of symptoms on admission but were not associated with other specific clinical features. CXCL13 could not be used as a prognostic marker for clinical recovery in pediatric LNB patients.

## Abbreviations

CNS: Central nervous system
CSF: Cerebrospinal fluid
CXCL13: Chemokine with CXC motif ligand 13
ELISA: Enzyme linked immunosorbent assay
EM: Erythema migrans
IgG: Immunoglobulin G
IgM: Immunoglobulin M
LB: Lyme borreliosis
LNB: Lyme neuroborreliosis
